# Robot movement planning for obstacle avoidance using reinforcement learning

**DOI:** 10.1038/s41598-025-17740-5

**Published:** 2025-09-12

**Authors:** Linda-Sophie Schneider, Junyan Peng, Andreas Maier

**Affiliations:** Pattern Recognition Lab, Friedrich-Alexander-University Erlangen-Nuremberg, Erlangen, Germany

**Keywords:** Computer science, Mechanical engineering

## Abstract

In modern industrial and laboratory environments, robotic arms often operate in complex, cluttered spaces. Ensuring reliable obstacle avoidance and efficient motion planning is therefore essential for safe performance. Motivated by the shortcomings of traditional path planning methods and the growing demand for intelligent automation, we propose a novel reinforcement learning framework that combines a modified artificial potential field (APF) method with the Deep Deterministic Policy Gradient algorithm. Our model is formulated in a continuous environment, which more accurately reflects real-world conditions compared to discrete models. This approach directly addresses the common local optimum issues of conventional APF, enabling the robot arm to navigate complex three-dimensional spaces, optimize its end-effector trajectory, and ensure full-body collision avoidance. Our main contributions include the integration of reinforcement learning factors into the APF framework and the design of a tailored reward mechanism with a compensation term to correct for suboptimal motion directions. This design not only mitigates the inherent limitations of APF in environments with closely spaced obstacles, but also improves performance in both simple and complex scenarios. Extensive experiments show that our method achieves safe and efficient obstacle avoidance with fewer steps and lower energy consumption compared to baseline models, including a TD3-based variant. These results clearly demonstrate the significant potential of our approach to advance robot motion planning in practical applications.

## Introduction

Multidegree-of-freedom robotic arms are an integral part of today’s industrial automation, manufacturing, and service applications. As these systems are deployed in increasingly complex and dynamic environments, achieving robust motion planning and reliable obstacle avoidance has become a critical challenge. In many practical scenarios, the robot must navigate through cluttered spaces and avoid collisions while optimizing its trajectory for efficiency and safety.

Efficient motion planning is essential for safely performing tasks in environments that are not only cluttered but also subject to dynamic changes. Conventional planning methods often struggle with adapting to unpredictable obstacles or high-dimensional configuration spaces. Traditional methods can be broadly categorized into searching-based, bionic and evolutionary, sampling-based, and gradient-based techniques. Searching-based algorithms, such as A*^1^, D*^2^, Dijkstra^3^, and simulated annealing^4^, are efficient in static, small-scale environments. However, they become impractical in large-scale, dynamic scenarios. Bionic methods like Genetic Algorithms (GA)^5^, Particle Swarm Optimization (PSO)^6^, Ant Colony Optimization (ACO)^7^, and fuzzy logic^8^ can handle more complex environments but often suffer from slow convergence and local optima.

Sampling-based approaches, such as Rapidly-Exploring Random Trees (RRT)^9^ and Probabilistic Roadmaps (PRM), can navigate intricate spaces but at the cost of computational complexity. Gradient-based methods, especially the artificial potential field (APF) method^10^, offer intuitive and computationally efficient solutions by modeling the workspace as a potential field. APF methods attract the end-effector toward the goal and repel it from obstacles, yet they are inherently vulnerable to local minima problems where the robot becomes trapped due to balanced attractive and repulsive forces^11,12^.

To address these limitations, hybrid strategies have been proposed. Integrating methods such as RRT with APF^9^, ACO with APF^13^, and Dynamic Window Approach (DWA) with GA^14^ have been explored. Researchers have also enhanced APF directly by modifying repulsive field functions or introducing auxiliary constructs. Cao^15^ proposed a velocity potential field (VPF) using robot velocity instead of distance, Flacco et al.^16^ leveraged repulsive vectors for collision avoidance, and Zhang et al.^17^ adjusted repulsive force functions to mitigate local minima.

In parallel, reinforcement learning (RL) methods, particularly Deep Deterministic Policy Gradient (DDPG), have emerged as robust tools for continuous control problems^18^. Early RL algorithms, such as Deep Q-Networks (DQNs)^19^, DDPG^18^, and SARSA^20^, enabled agents to learn optimal policies by interacting with their environment. Recent advancements have further expanded these techniques. Chen^21^ utilized DDPG, TD3, and SAC effectively in robotic tasks, while Imtiaz^22^ demonstrated the versatility of PPO in fruit-picking applications. Improvements such as the multi-critic mechanism by Sze^23^ and refined reward functions by Li et al.^24^ have enhanced policy evaluation and APF optimization, respectively. Hybrid approaches combining RL with traditional methods, like RRT with TD3^25^, DWA with Q-learning^26^, and PRM with RL^27^, further illustrate the complementary potential of these strategies.

Despite these advancements, critical challenges persist, notably the local minima issue in APF methods and the sensitivity of RL algorithms to reward function design. The APF method’s vulnerability to local minima arises when nearby obstacles counterbalance the attractive goal force, trapping the robot prematurely. Conversely, DDPG and similar RL methods can learn complex continuous action policies but often struggle with limited or misleading rewards in complex environments.

Our proposed approach integrates a modified APF mechanism within the DDPG framework, combining APF s structural guidance to alleviate local minima and DDPG s adaptive learning capabilities. Specifically, we enhance the reward signal with APF-derived attractive and repulsive components. Our state space includes both joint angles and end-effector positions, and actions comprise fine-grained joint rotations. This hybrid strategy enables efficient obstacle avoidance and optimized motion planning in a fully continuous state and action space, in contrast to the discrete formulations used in some prior work.

While some prior approaches combine APF with RL methods such as Q-learning or PPO^28,29^, they typically rely on discretized state spaces, task-specific constraints, or lack the fine-grained control needed for articulated robotic arms. Others focus primarily on mobile robots, where the configuration space is of lower dimensionality and local minima are easier to mitigate. In contrast, our approach is explicitly designed for continuous 6-DOF robotic arms operating in 3D space, where both configuration complexity and the risk of local minima are substantially higher. Moreover, our APF-informed reward function is designed to remain informative throughout training, facilitating faster convergence and more stable behavior across environments of varying complexity.

The contributions of our work are threefold: First, we present a novel APF-DDPG hybrid framework that combines the advantages of traditional and learning-based methods in the continuous setting. Second, we propose an improved reward mechanism explicitly designed to mitigate local minima. Finally, we validate our framework through extensive experiments across diverse environments, demonstrating significant improvements in convergence, solution quality, and collision avoidance compared to baseline models. Our findings provide robust solutions for real-world robotic arm motion planning.

## Methodology

### Robot kinematic model

In this work, we employ a standard 6-degree-of-freedom industrial manipulator^30^ as our experimental platform. The kinematic model of the robot is derived using the standard Denavit–Hartenberg (DH) convention. According to this convention, each joint is assigned a coordinate frame, enabling the definition of spatial transformations between consecutive joints.

Using these DH parameters, we calculate the forward kinematics to determine the robot configuration. Each transformation matrix ${}^{i-1}\mathbf{T}{i}$ from joint $i-1$ to joint *i* is defined as

1 $$\begin{aligned} {}^{i-1}\mathbf{T}_{i} = \begin{bmatrix} \cos \theta _i & \quad -\sin \theta _i \cos \alpha _i & \quad \sin \theta _i \sin \alpha _i & \quad a_i \cos \theta _i \\ \sin \theta _i & \quad \cos \theta _i \cos \alpha _i & \quad -\cos \theta _i \sin \alpha _i & \quad a_i \sin \theta _i \\ 0 & \quad \sin \alpha _i & \quad \cos \alpha _i & \quad d_i \\ 0 & \quad 0 & \quad 0 & \quad 1 \end{bmatrix}. \end{aligned}$$

The complete forward kinematic transformation from the base frame to the end-effector frame is obtained by multiplying these matrices sequentially:

2 $$\begin{aligned} {}^{0}\mathbf{T}{6} = \prod {i=1}^{6} {}^{i-1}\mathbf{T}_{i}. \end{aligned}$$

From ${}^{0}\mathbf{T}_{6}$, the end-effector position (*x*, *y*, *z*) is extracted and used for collision detection and distance computations in the obstacle avoidance algorithm. By incorporating forward kinematics directly into our methodology, we simplify computational requirements while ensuring accurate spatial data for our reinforcement learning framework.

### Continuous obstacle avoidance with APF-DDPG framework

In this work, we propose an obstacle avoidance approach that integrates APF with DDPG reinforcement learning^31^. This integration addresses critical limitations of each method individually. APF methods often encounter local minima problems, particularly when attractive and repulsive forces oppose each other^32^. Pure DDPG methods, in contrast, typically require extensive exploration and lack direct spatial information, potentially slowing convergence and complicating navigation. The combined APF-DDPG framework thus leverages structured spatial information from APF and the robust exploratory learning ability of DDPG, facilitating efficient and reliable obstacle avoidance in continuous state-action scenarios.

#### Problem definition and state-action representation

We define the obstacle avoidance problem as a Markov Decision Process (MDP), described by a state space $\mathscr {S}$, action space $\mathscr {A}$, and a reward function *R*(*s*, *a*). The continuous state vector $s \in \mathbb {R}^{9}$ comprises the robot s six joint angles $(\theta _1,\dots ,\theta _6)$ and the end-effector’s Cartesian coordinates (*x*, *y*, *z*) computed from forward kinematics. The action vector $a \in \mathbb {R}^{6}$ specifies incremental joint angle adjustments within the interval of $[-0.5^\circ , 0.5^\circ ]$ per timestep, allowing smooth and controlled movements.

#### APF-DDPG framework

**Fig. 1:**
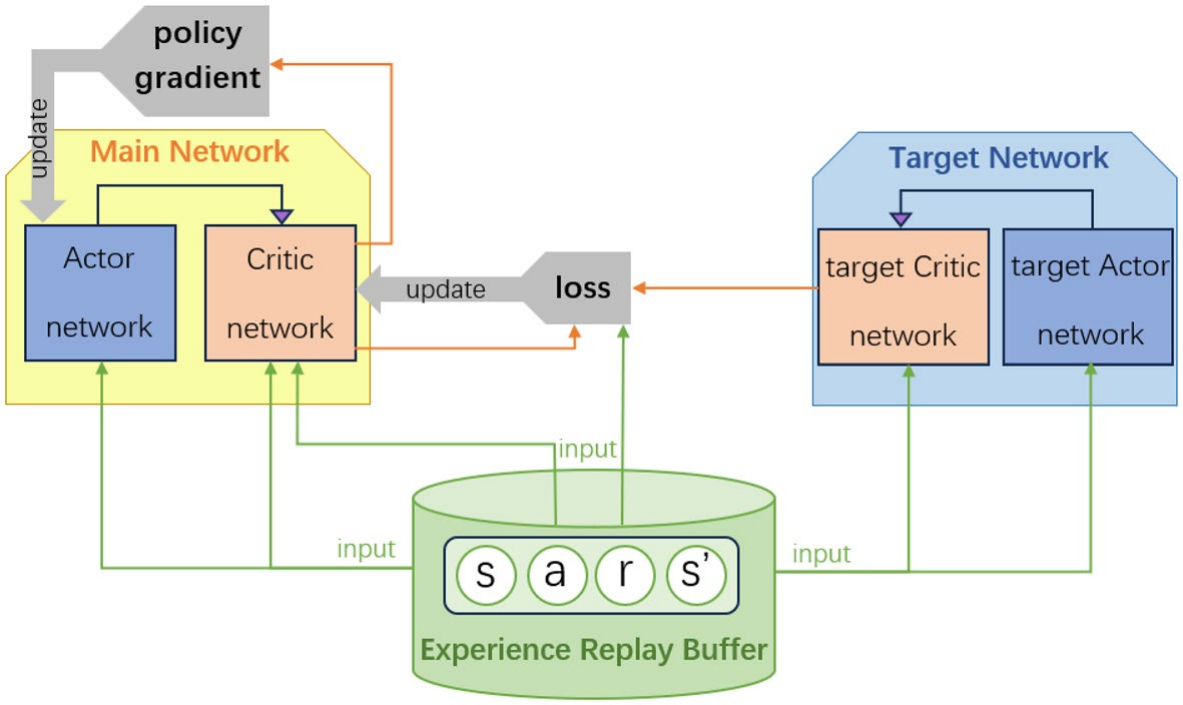
Integrated APF-DDPG framework for continuous obstacle avoidance. The main networks (actor and critic) use spatial information from APF directions to guide policy updates. Target networks stabilize training. Experiences $(s,a,r,s')$ are sampled from the replay buffer to decorrelate training updates.

The APF-DDPG framework consists of actor and critic neural networks, their respective target networks, and an experience replay buffer for stable training, as illustrated in Fig. 1. At each timestep, the actor network outputs continuous incremental actions given the current state, which are then executed by the robot. The critic network evaluates state-action pairs by estimating the expected cumulative rewards (Q-values).

The networks are trained using mini-batches sampled from the replay buffer, where each stored experience tuple $(s,a,r,s')$ captures interactions with the environment. To ensure stable training and smooth convergence, we follow^32^ and employ soft target network updates, defined by

3 $$\begin{aligned} \theta ' \leftarrow \tau \theta + (1 - \tau )\theta ', \quad 0 < \tau \ll 1, \end{aligned}$$

where $\theta '$ are target network parameters, $\theta$ are main network parameters, and $\tau$ being the smoothing coefficient.

#### APF integration and compensation mechanism

We integrate APF into our DDPG framework to explicitly inform the actor network of desirable spatial directions. To do this, we first recall the standard artificial potential field definition^10^

$$U_{\textrm{att}}(q) = \tfrac{1}{2}\,k_{\textrm{att}}\bigl \Vert q - q_{\textrm{goal}}\bigr \Vert ^2, \qquad U_{\textrm{rep}}(q) = {\left\{ \begin{array}{ll} \tfrac{1}{2}\,k_{\textrm{rep}}\Bigl (\tfrac{1}{\rho (q)} - \tfrac{1}{\rho _0}\Bigr )^2, & \quad \rho (q)\le \rho _0,\\ 0, & \quad \rho (q)>\rho _0, \end{array}\right. }$$

where $q$ is the end-effector position, $q_{\textrm{goal}}$ the goal position, $\rho (q)$ the distance to the nearest obstacle, and $\rho _0$ its influence radius. Forces arise as the negative gradients of these potentials:

$$F_a(q) = -\nabla U_{\textrm{att}}(q), \qquad F_r(q) = -\nabla U_{\textrm{rep}}(q).$$

The net direction of the APF is then $F_\textrm{net} = F_a + F_r$. However, when $F_a$ and $F_r$ directly oppose each other, the robot can become trapped in a local minimum ($\Vert F_\textrm{net}\Vert \approx 0$).

To systematically escape such traps, drawing inspiration from the classical BUG principle^33^, we add a small orthogonal perturbation. Specifically, we define

4 $$\begin{aligned} F_\perp \;=\; \frac{F_a \times F_r}{\bigl \Vert F_a \times F_r\bigr \Vert }, \qquad F_{\textrm{adjusted}} \;=\; F_a + F_r + \gamma \,F_\perp , \quad \gamma \ll 1. \end{aligned}$$

**Fig. 2:**
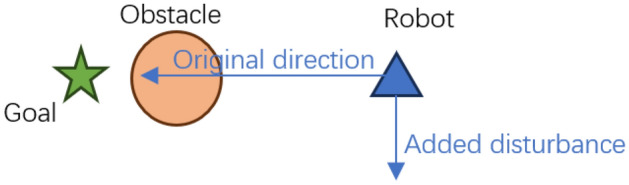
Illustration of perpendicular compensation in APF direction. When repulsive and attractive forces nearly oppose each other, a perpendicular correction is introduced, steering the robot away from local minima.

Because $F_\perp$ is normal to the plane spanned by $F_a$ and $F_r$, this small perturbation guarantees movement off any planar saddle without significantly diverting the primary APF direction. The adjusted force $F_{\textrm{adjusted}}$ is then provided to the actor network at each time step, biasing policy updates toward collision-free, goal-directed motion. The compensation mechanism is schematically illustrated in Fig. 2.

#### Reward function design

The reward function is carefully designed to balance obstacle avoidance and goal-oriented behavior by combining two components: proximity to the goal and directional alignment with APF guidance. Formally, we write

5 $$\begin{aligned} R(s,a) = -\lambda \,\textrm{distanceGE} + \mu \bigl (\cos (\sigma ) - 1\bigr ), \end{aligned}$$

where $\textrm{distanceGE}$ is the Euclidean distance between the end-effector and the goal in Cartesian space, explicitly encouraging movements toward the goal. The term $\cos (\sigma )\in [-1,1]$ measures the alignment between the executed displacement vector $\overrightarrow{\textrm{EE}}$ and the APF-suggested vector $\overrightarrow{\textrm{APF}}$, computed as

6 $$\begin{aligned} \cos (\sigma ) = \frac{\overrightarrow{\textrm{EE}}\cdot \overrightarrow{\textrm{APF}}}{\Vert \overrightarrow{\textrm{EE}}\Vert \;\Vert \overrightarrow{\textrm{APF}}\Vert }. \end{aligned}$$

We choose $\mu >1$ so that $\mu (\cos \sigma -1)\le 0$ with equality only when $\cos \sigma =1$, ensuring every deviation from perfect alignment incurs a negative reward and thereby shaping the agent to follow efficient, APF-consistent motions. To incorporate safety constraints we distinguish collision scenarios by using separate distance-weighting parameters, defining

7 $$\begin{aligned} R(s,a)= {\left\{ \begin{array}{ll} -\lambda _{c}\,\textrm{distanceGE} + \mu (\cos (\sigma ) - 1), & \quad \text {if collision},\\ -\lambda _{nc}\,\textrm{distanceGE} + \mu (\cos (\sigma ) - 1), & \quad \text {otherwise}, \end{array}\right. } \end{aligned}$$

where $\lambda _{c}>\lambda _{nc}$ is chosen based on the ratio between the workspace size and the robot arm length so that the distance penalty scales appropriately with the environment s spatial dimensions relative to the manipulator. We allow collisions during training rather than terminating episodes to promote extensive exploration of the state action space and to enable the agent to learn both avoidance and recovery strategies. Unlike pure APF methods that stall in local minima, our formulation maintains a negative distance term $R_{\textrm{dist}} = -\lambda \,\textrm{distanceGE}$ until the goal is reached. We apply a per-step penalty to prevent hesitation or oscillation within shallow basins. We allow non-terminating collisions with a larger penalty $\lambda _{c}$, encouraging risky detours and subsequent recovery to uncover escape pathways. We use the alignment term $R_{\textrm{align}} = \mu (\cos \sigma - 1)\le 0$ to penalize moves that conflict with APF guidance. As a result, the agent converges on efficient, collision-free trajectories.

## Experimental setup

### Design of task environments

**Fig. 3:**
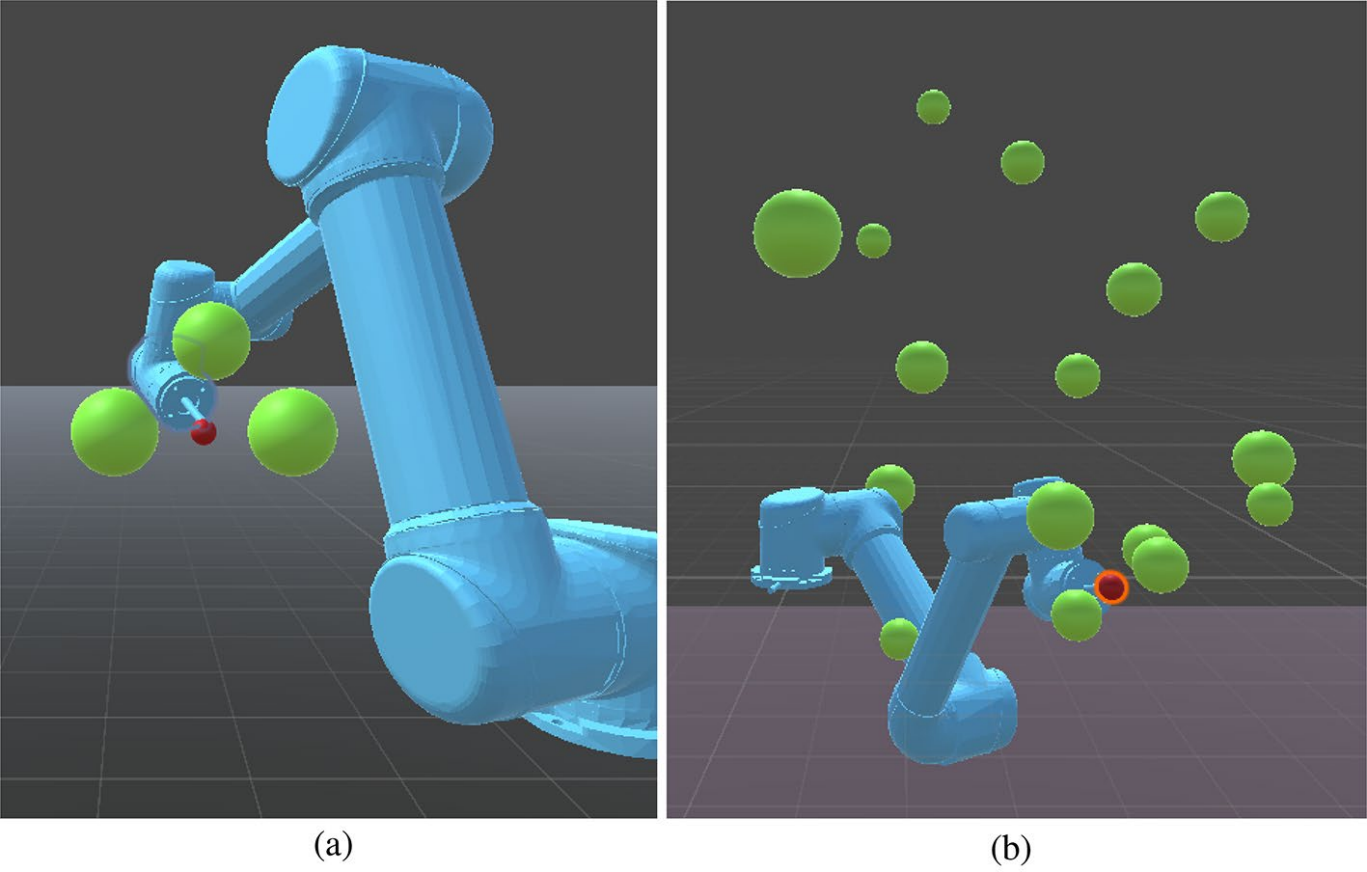
Left: Example of a simple task environment containing fewer than ten spherical obstacles positioned to create local optima traps. Right: Example of a complex task environment, illustrating increased obstacle density and strategic placement to enhance task difficulty. (These images were made with Unity-2022.3.18f1, https://unity.com/de).

Due to the absence of specialized datasets for robotic arm path planning and obstacle avoidance, we developed a series of custom environments to simulate diverse real-world scenarios. Each obstacle within these environments is assigned a region characterized by a specific repulsion scaling factor, resulting in localized repulsive fields that directly affect the agent s trajectory planning. All environments share a common workspace dimension of $10 \times 10 \times 10$ units. Spherical obstacles are placed strategically and vary in radius from $0.2$ to $0.5$ units, while the robotic arm itself, when fully extended, approximates a cylinder of length $10$ units with a radius of $0.1$ units. We designed two distinct categories of task environments to rigorously evaluate the proposed approach:

*Simple task environments* These environments include fewer than ten spherical obstacles strategically arranged to create deliberate local optima traps, as depicted in Fig. 3a. These scenarios provide baseline conditions to assess fundamental obstacle avoidance capabilities.

**Fig. 4:**
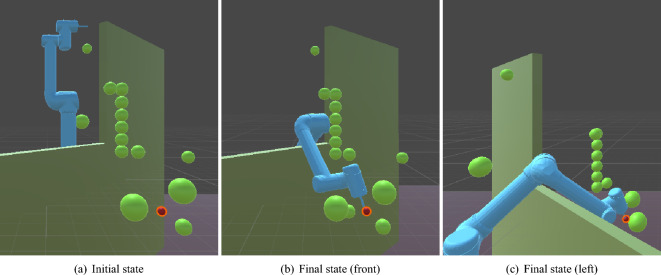
A complex task environment featuring cuboid wall obstacles, significantly restricting movement space compared to spherical obstacles. (These images were made with Unity-2022.3.18f1.

*Complex task environments* These scenarios incorporate a higher number and strategic complexity of obstacles, significantly increasing the likelihood of collisions and entrapment in local optima. Obstacles are placed near the initial position to hinder initial movements and along intended trajectories to elevate the complexity of the planning problem. Figure 3b illustrates typical complex environments. An additional challenging scenario includes environments featuring cuboid wall obstacles (0.5 units thickness, 5 units width, and 10 units height), as shown in Fig. 4. The initial placement (Fig. 4a), along with the front (Fig. 4b) and side (Fig. 4c) views of the final state, emphasize how the dimensions of the wall impose substantial restrictions on the agent’s movement, covering approximately 30–40% of the width of the workspace and 50% height, providing a strict test for obstacle avoidance capabilities.

The experimental setup comprises a total of 30 simple and 30 complex environments. Additionally, multi-goal trajectory planning tasks were designed, where the final state of one iteration serves as the initial state of the next. Task completion is achieved when the end-effector reaches the goal within a 0.1-unit tolerance, optimizing for computational efficiency and practical accuracy.

### Simulation environment and implementation

For intuitive visualization and precise control of the robotic agent, Unity, a real-time 3D development platform, is employed. Control scripts are implemented using C# to directly interact with the Unity simulation environment. Experiments were conducted on a computing system with an 11th Gen Intel(R) Core(TM) i5-11320H processor and NVIDIA GeForce MX450 GPU. The reinforcement learning framework was implemented using PyTorch 2.0.1. Detailed hardware and software configurations are summarized in Table 1.

**Table 1:** Hardware and software configuration.

Item	Specification
CPU	11th Gen Intel(R) Core(TM) i5-11320H @ 3.20 GHz 2.50 GHz
GPU	NVIDIA GeForce MX450
PyTorch	2.0.1
Unity	2022.3.18f1

### Baseline methods for comparison

The choice of baseline methods was motivated by their contrasting characteristics, strengths, and limitations relevant to robotic arm path planning. To fully evaluate the effectiveness of the proposed DDPG-APF approach, we compare with the following baselines:

- A* Search (Non-RL) A classical best-first search algorithm operating in a discretized joint-action space (rotations of − 1 , 0 , + 1 per joint). A* is run without a step limit, relying on an admissible and consistent heuristic to guarantee optimality in discrete settings^34^. This method serves as a non-learning baseline, highlighting the challenges of discretization and potential local-optima loops in our custom environments.
- *Pure-DDPG* A standard Deep Deterministic Policy Gradient model without Artificial Potential Field guidance, using only collision penalties and distance-to-goal in its reward function. This baseline isolates the benefit of APF integration and demonstrates the intrinsic performance limitations and convergence challenges faced by reinforcement learning without heuristic guidance.
- *TD3-APF* Twin Delayed DDPG with the same APF-based reward as our DDPG-APF agent. TD3 employs dual critics and delayed actor updates to address DDPG’s overestimation bias^35^, enabling a direct comparison under identical reward and network settings to assess whether these algorithmic improvements provide tangible benefits in complex, continuous task spaces.

All reinforcement learning methods share identical network architectures and training regimens, ensuring a fair evaluation of feasibility, optimality, computational efficiency, and convergence behavior. Together, these methods provide a comprehensive performance context, ranging from classical planning paradigms to modern deep reinforcement learning techniques.

## Results

We evaluated the performance of four distinct algorithms: DDPG-APF, Pure-DDPG, TD3-APF, and the classical A* search algorithm. This evaluation was conducted across tasks of varying complexity, focusing specifically on path optimality, solution feasibility, computational efficiency, and learning dynamics. All RL algorithms were trained under identical hyperparameters, explicitly detailed in Table 2.

**Table 2:** Chosen parameters for the RL algorithms.

Parameter	Value
Update frequency	Every 50 steps
Batch size	256
Replay buffer size	$1\times 10^{5}$
Discount factor ($\gamma$)	0.98
Learning rate	$1\times 10^{-3}$
TD3 soft-update rate	$5\times 10^{-3}$

**Fig. 5:**
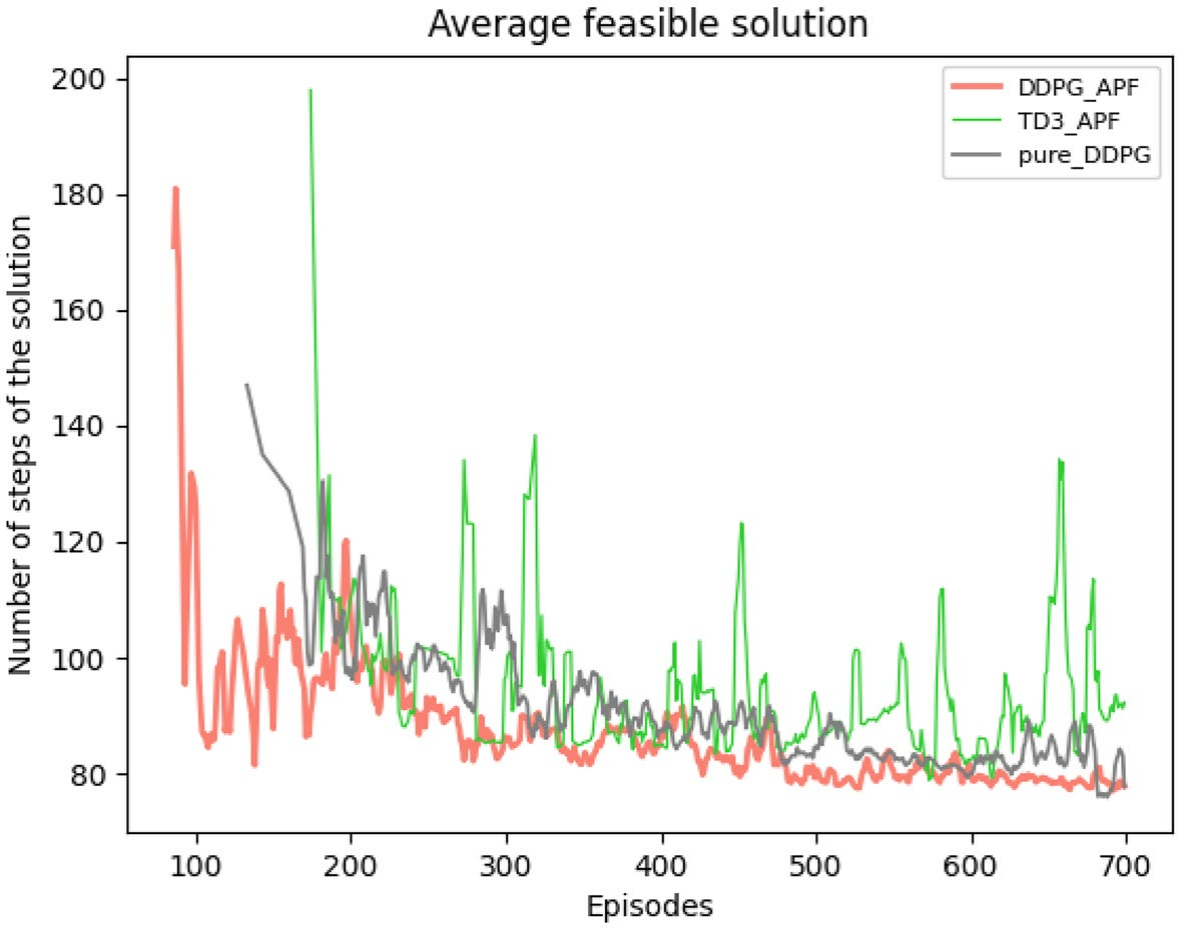
Feasible solution evaluation illustrating episodes required for finding the first feasible path. DDPG-APF identifies feasible paths sooner and more consistently than other methods, reflecting superior stability and efficiency.

Initially, we analyzed training dynamics and convergence characteristics. The progression of best solutions, quantified by the number of steps required to reach the goal, revealed clear distinctions among the evaluated methods. Initially, all algorithms maintained a step count at the maximum threshold of 200 steps, indicating the absence of early feasible solutions. Upon discovering feasible solutions, the DDPG-APF algorithm exhibited notably faster convergence, consistently achieving shorter paths at an earlier stage, as demonstrated in Fig. 5.

**Fig. 6:**
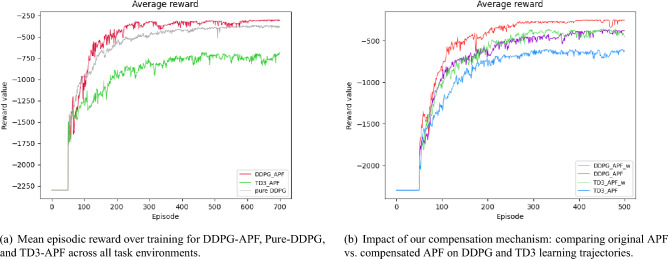
Comparison of the presented reward functions: (**a**) overall model performance across all tasks; (**b**) sensitivity to reward-function design.

While the episode count to first feasible solution quantifies *when* each agent begins to find successful paths, analyzing the evolution of the average reward provides deeper insights into *how effectively* each policy learns over time. Specifically, tracking rewards captures both the stability of learning dynamics and the efficiency with which algorithms balance exploration, collision avoidance, and path-length minimization (see Fig. 6). Figure 6a illustrates the overall average reward curves across all task environments. Here, DDPG-APF consistently achieves a higher average reward compared to Pure-DDPG and TD3-APF, signaling more efficient policy improvements and a superior exploration-exploitation trade-off. In contrast, Pure-DDPG demonstrates a smoother but slower progression, reflecting a stable yet less efficient learning pattern, whereas TD3-APF displays noticeable fluctuations, likely resulting from increased complexity associated with its dual-critic network structure.

We further assessed the sensitivity of the RL algorithms to variations in reward function design. Two distinct reward functions were tested: the original APF method without modifications and our proposed compensated APF reward function. Reward curves averaged across all tasks, displayed in Fig. 6b, demonstrated that DDPG-APF maintained stable performance regardless of reward function variations. Conversely, TD3-APF exhibited greater sensitivity, likely due to additional complexity in its dual-network architecture aimed at mitigating value function overestimation.

In comparing RL methods to classical non-learning approaches, we evaluated the A* search algorithm, which operates using discrete action spaces and heuristic search. A comprehensive summary of key performance metrics across all 60 scenarios is provided in Table 3. Despite having unlimited search steps per episode, A* exhibited significantly lower success rates compared to the RL-based methods. Specifically, DDPG-APF demonstrated superior performance in average steps, success rate, and episodes required to achieve first success. Moreover, a dispersion analysis shows that DDPG-APF s variability in average steps is roughly half that of Pure-DDPG and one-third that of TD3-APF, and its variability in episodes to first success is about 45% lower than Pure-DDPG and over 65% lower than TD3-APF clear evidence of its consistency across diverse scenarios. The results highlight the advantages of continuous action adjustments offered by DDPG-APF, particularly in scenarios sensitive to local optima.

Further evaluation against other RL metrics, such as time per episode and stability of solution improvement, reinforces the robustness of the DDPG-APF method. DDPG-APF consistently outperformed Pure-DDPG and TD3-APF across all metrics, particularly in computational efficiency (Time/Episode), indicating faster convergence and more effective exploration strategies. Notably, DDPG-APF reduces time-per-episode variability by approximately 25% relative to TD3-APF, underscoring its reliable runtime behavior. TD3-APF, while theoretically robust, showed notably higher variability and longer episodes to achieve first success, highlighting challenges associated with its dual-critic architecture.

**Table 3:** Summary of performance metrics (mean ± std over 3 runs for all 60 scenarios).

Agent	Avg. steps	Success rate (%)	Episodes to first success	Time/episode (s)
DDPG-APF	72.5 ± 17.8	91.6	168.18 ± 81.8	1.24 ± 0.31
Pure-DDPG	99.9 ± 35.6	83.3	279.3 ± 164.6	1.36 ± 0.21
TD3-APF	121.6 ± 57.7	76.7	324.7 ± 248.8	1.635 ± 0.416
A* Search	382.9 ± 271.3	63	382.9 ± 271.3	1.71 ± 0.42

Computational efficiency was further analyzed by measuring average runtime per episode across tasks with different complexity levels. Table 4 summarizes these results, showing DDPG-APF consistently offering computational advantages over TD3-APF and Pure-DDPG.

**Table 4:** Average episode runtime (seconds) for tasks differentiated by complexity. A* is computed as average time/step.

Agents	Simple tasks	Complex tasks without wall	Complex tasks with wall
DDPG-APF	1.051	1.425	1.460
Pure-DDPG	1.225	1.526	1.515
TD3-APF	1.543	1.833	1.859
A* Search	1.375	1.924	2.067

These runtime trends align well with our task complexity classification. Average episode runtime increases steadily from simple tasks through complex tasks without wall to complex tasks with wall. The additional runtime in scenarios containing walls reflects the greater number of collision checks and potential field updates required to navigate planar obstacles. This monotonic increase in computational load confirms that our categorization of simple and hard tasks is reasonable and that wall environments rightly belong in the hard category.

To identify the precise contributors to these runtimes we divide each episode into agent runtime and environment preparation runtime. Agent runtime comprises action selection at every step and network updating every fifty steps and is dominated by reinforcement learning computations. Environment preparation updates the environment state around the agent and is driven primarily by artificial potential field calculations. For the DDPG-APF agent action selection requires around 0.5 ms network updating approximately 240 ms and environment preparation about 2 ms. For the TD3-APF agent action selection requires around 0.7 ms network updating approximately 280 ms and environment preparation about 2 ms.

Across all analyzed metrics, DDPG-APF consistently outperforms both RL baselines and the classical A* method, validating the benefit of APF-guided reward shaping in continuous control settings.

## Discussion

The APF-DDPG framework illustrates how combining structural guidance with adaptive learning can yield both stability and efficiency in robotic motion planning. Our experiments highlight clear benefits over classical approaches and pure reinforcement learning, but they also expose new compromises that arise from this hybrid design. In the following, we reflect on these strengths and weaknesses, emphasizing the trade-offs between guidance and exploration, efficiency and flexibility, as well as simulation and real-world applicability.

### Continuous state-action formulation

Using a continuous state-action formulation provides clear benefits over discretized or inverse kinematics-based methods. Continuous incremental actions allowed the agent to adapt flexibly to dynamic obstacle configurations, enabling responsive trajectory adjustments. At the same time, the use of forward kinematics reduced computational complexity by avoiding costly inverse kinematics calculations, resulting in shorter episode runtimes compared to baseline methods. A drawback is that this formulation inherently enlarges the search domain, requiring careful reward shaping and parameter tuning to maintain stability; without strong guidance, training in such a continuous space may become less robust.

### Learning efficiency

The framework demonstrated improved learning efficiency, with faster convergence and more stable reward trajectories. The dense reward function provided continuous and informative feedback, facilitating effective optimization and reducing the number of collision trials. On the other hand, this reliance on a dense reward structure represents a limitation: if such shaping is not available, or must be inferred from noisy real-world signals, efficiency and stability may degrade substantially.

### APF integration and trade-offs

Integrating a modified APF within the DDPG framework shaped the reward structure into a smoother potential field that continuously guided the agent toward the goal while repelling it from obstacles. This reduced fluctuations, stabilized learning, and biased exploration toward safer paths, improving sample efficiency and runtime compared to Pure-DDPG. At the same time, this integration introduced important trade-offs. The strong directional bias of APF may suppress the discovery of unconventional but potentially more efficient trajectories, and the perpendicular compensation term, while effective in our experiments, could in principle induce oscillatory motion in narrow passages. Moreover, the continuous computation of potential field forces and collision checks adds a runtime overhead of approximately 2 ms per step. Our experiments indicate that this cost is outweighed by fewer detours, reduced collisions, and faster convergence, yet its impact under noisy sensing or dynamic obstacles remains an open question. Together, these findings highlight both the stabilizing benefits and the potential risks of APF guidance.

### Observed limitations

Our experiments revealed two concrete shortcomings of the current implementation. First, the framework lacks adaptability in adjusting step limits to different task complexities, which led to degraded performance in harder scenarios. Second, the method struggled with smooth transitions in multi-goal trajectory planning, limiting its effectiveness in sequential manipulation tasks. These weaknesses point to the need for adaptive step management and hierarchical or goal-decomposition strategies in future work.

### Dataset limitations and evaluation fairness

The dataset and evaluation design impose further limitations. All environments were manually created to challenge the robot arm, which restricts generalizability and prevents a definitive claim that APF-DDPG consistently outperforms other agents. The absence of standardized benchmarks for manipulator path planning further complicates comparisons across studies. Moreover, although oscillatory behavior was not observed in our fewer than 100 task instances, it cannot be excluded on larger or more uniform benchmarks. Parameter tuning across algorithms is another source of uncertainty: TD3, for example, did not achieve the expected improvements over DDPG, possibly because its double actor-critic structure was less advantageous in our task setup. These findings suggest that notions such as overestimation in reinforcement learning should be reconsidered in context, as optimistic Q-value estimates in DDPG may sometimes aid exploration rather than hinder it.

### Application niches

The APF-DDPG framework is particularly suited to industrial scenarios where manipulators must operate safely in highly cluttered but largely static workcells. Examples include dynamic pick-and-place in warehouses or laboratory automation tasks, where dense static obstacles constrain free exploration and safety demands are high. In such settings, the spatial guidance of APF can provide reliable safety margins, while reinforcement learning enables adaptability to variations in object placement. In contrast, long-range navigation or human robot collaboration with highly dynamic obstacles may be less suited, since strong APF bias could hinder the discovery of unconventional solutions. Short- to mid-range manipulation in dense, safety-critical environments therefore appears to be the most promising niche for our approach.

### Sim-to-real considerations

Finally, the transferability of our findings from simulation to real-world deployment remains a challenge. The modeling assumptions made in our setup are fragile under realistic conditions. For instance, the UR5 model neglects actuator dynamics, backlash, and latency, which influence fine-grained incremental control. Likewise, APF forces were computed under idealized, noise-free conditions, whereas real-world sensing inevitably introduces uncertainty in obstacle localization. The obstacles themselves were represented by simple geometric primitives, in contrast to the irregular and textured shapes encountered in practice. These simplifications suggest that APF-DDPG may be particularly sensitive to sensor noise and geometric mismatches. Future work should therefore investigate robustness under imperfect perception and more realistic robot models to narrow the sim-to-real gap.

## Conclusion

In this paper, we presented a reinforcement learning framework integrating a modified APF method with DDPG for robotic arm motion planning in continuous 3D environments. Our approach addresses the local optimum issues inherent in traditional APF methods through a tailored reward mechanism. Experimental results demonstrated that our method can find feasible solutions in fewer episodes and typically achieves more optimal paths compared to baseline models. Although APF introduces extra computation per step, in practice this overhead is outweighed by more efficient trajectories, leading to faster overall execution.

The findings suggest that APF-based guidance not only accelerates convergence but also improves computational efficiency, making it suitable for complex obstacle avoidance tasks. However, the method’s current limitations, particularly the inability to automatically adjust step limits for varying complexities and difficulties in smoothly handling multi-goal trajectory planning, highlight important areas for further improvement.

Future research should explore adaptive parameter tuning methods to enhance performance consistency across different task complexities. Additionally, developing more efficient algorithms for trajectory planning involving multiple sequential goals and extending the framework to support multi-agent cooperation and dynamic obstacle avoidance are promising directions. Ultimately, addressing these challenges will significantly narrow the gap between theoretical advances and practical robotic applications.

## Data Availability

The data and code that support the findings of this study are available at the following GitHub repository: https://github.com/JunyanPeng330/RobotPlanning. For further information or access to additional data, please contact the corresponding author.
